# The use of observed-to-expected analyses as a signal detection tool in COVID-19 vaccine safety surveillance: lessons learned from an industry perspective

**DOI:** 10.3389/fdsfr.2025.1650992

**Published:** 2025-12-01

**Authors:** Laurence Serradell, Antonella Fretta, Jenny Nachbar, Jill Dreyfus, Diana Garofalo, Arianna Lucini, Susan Mather, Daina Esposito, Anne-Laure Chabanon, Vincent Bauchau, Sarah Sellers

**Affiliations:** 1 Epidemiology and Benefit Risk, Patient Safety and Pharmacovigilance, Sanofi, Lyon, France; 2 Worldwide Medical and Safety, Pfizer Inc., Milan, Italy; 3 Global Vaccine Safety, Novavax, Inc., Gaithersburg, MD, United States; 4 Global Medical Epidemiology, Pfizer, Inc., New York, NY, United States; 5 Worldwide Medical and Safety, Pfizer Inc., Collegeville, PA, United States; 6 Global Safety Epidemiology, Moderna Inc., Cambridge, MA, United States; 7 Patient Safety and Pharmacovigilance, Sanofi, Lyon, France; 8 GSK, Wavre, Belgium

**Keywords:** observed-to-expected, adverse event of special interest, COVID-19 vaccine, signal detection, signal refinement

## Abstract

During the pandemic, the accelerated review and authorization of coronavirus disease 19 (COVID-19) vaccines by regulatory authorities elicited the need for rapid and thorough worldwide signal detection and evaluation. To meet this need, the European Medicines Agency and other health authorities expected that, in addition to routine signal detection, COVID-19 vaccine manufacturers should leverage observed-to-expected (O/E) analyses unconventionally as a quantitative method for signal detection of adverse events of special interest (AESIs). The objective of O/E analyses in vaccine signal detection was to determine if AESIs were occurring at a higher-than-expected rate in the vaccinated population in comparison with an unexposed population. The use of O/E was intended to mitigate the challenge of analyzing large volumes of individual case safety reports (ICSRs) received over a very short period following mass vaccination campaigns. The “Beyond COVID-19 Monitoring Excellence” (BeCOME) initiative, a non-competitive voluntary initiative launched in 2022 by COVID-19 vaccine Marketing Authorization Holders (MAHs) and key stakeholders, was established to align systems, enhance processes, and foster innovation in post-marketing vaccine monitoring, building on lessons from the pandemic. A dedicated working group was created to review and share MAHs’ experience on O/E analyses used as an additional tool for signal detection during the COVID-19 pandemic. This review presents the industry perspective on using O/E analyses for COVID-19 vaccine signal detection, including challenges and limitations encountered, and proposes best practices for future improvement. Despite the priority and resources devoted to O/E analyses, no *de novo* signals resulting in the identification of safety concerns were detected using this methodology during the COVID-19 pandemic. O/E analyses are most useful when source data are accurate and there is a high level of confidence in the assumptions and parameters used. In the context of the COVID-19 pandemic, confidence in certain assumptions and parameters was low, limiting the value of O/E analyses in signal detection. Nevertheless, O/E analyses applied for signal refinement, as traditionally used, proved to be useful. Industry experiences support maintaining O/E analyses as a tool for signal refinement and standardizing methodological approaches as much as possible to enhance its future application and comparability across stakeholders.

## 1 Introduction

Due to the significant morbidity and mortality of coronavirus disease 19 (COVID-19) caused by severe acute respiratory syndrome coronavirus 2 (SARS-CoV-2) and the lack of effective treatments, vaccine developers and regulatory authorities worked in an unprecedented manner to facilitate the rapid availability of vaccines. Typically, the development and approval processes for vaccines require vaccine developers to complete clinical trials and then prepare dossiers of trial data for submission to regulatory authorities, who conduct a review process that can take several years. For the COVID-19 vaccines, the regulatory review process was conducted under emergency use authorization procedures enabling the submission of clinical trial data as it accrued and at trial completion. This required precise coordination and around-the-clock commitment by both regulatory authorities and vaccine developers. In addition, vaccine advisory committees, such as the Advisory Committee on Immunization Practices (ACIP) in the United States (US), convened more frequently than usual to review available data, hold public discussions to characterize the evolving pandemic and to provide recommendations for vaccines use. As a result of this concerted and coordinated effort, the first COVID-19 vaccines became available for use through conditional approvals less than a year after the COVID-19 public health emergency was declared.

Prior to approval, anticipated mass vaccination campaigns prompted the European Medicines Agency (EMA) to publish a specific guidance on post-marketing surveillance requirements in the European Union (EU) (“EMA Consideration on core requirements for RMPs of COVID-19 vaccines” (European Medicines Agency, 2022a)). This guidance acknowledged that routine signal detection methods and practices might be insufficient to quickly and efficiently examine the high volumes of individual case safety reports (ICSRs)^1^ expected to be reported to regulatory authorities and Marketing Authorization Holders (MAHs) following mass vaccination campaigns. Among other methods to support signal detection activities, MAHs were required to leverage observed to expected (O/E) analyses as a quantitative method for signal detection of adverse events of special interest (AESIs^2^), regardless of the vaccine platform technology used for their respective marketed vaccines. O/E analysis is a statistical tool that compares the rate of an event in a population receiving the vaccine of interest (i.e., the observed rate) with the rate in a population without exposure to the vaccine (i.e., the expected rate). This application of O/E analysis for signal detection was unconventional, given its traditional use in refining and evaluating signals detected through other methods, such as review of individual or aggregate adverse event (AE) reports from clinical trials and post-marketing use, disproportionality analyses of AEs, and review of safety information from literature (Mahaux et al., 2016).

The “Beyond COVID-19 Monitoring Excellence” (BeCOME) initiative, a non-competitive voluntary initiative launched in 2022 by COVID-19 vaccine MAHs and other key stakeholders, was established to enhance systems, align procedures, and foster innovation in post-marketing vaccine monitoring, based on real-time learnings across the pandemic. A dedicated working group was created to review and share MAHs’ experience regarding O/E analyses used as a tool for AESIs signal detection during the COVID-19 pandemic. This review presents the experience and perspectives of COVID-19 vaccine MAHs on using O/E analyses for AESIs signal detection during the COVID-19 pandemic, including limitations encountered and proposals for future improvements. Insights from vaccine manufacturers foster a deeper understanding of the challenges and approaches related to vaccine safety monitoring during pandemics and are expected to support improvement of the relevant systems for determining vaccine benefit risk assessment, both in pandemic and non-pandemic scenarios (Bauchau et al., 2023; Bauchau et al., 2025).

## 2 Purpose and methodology of O/E analyses

O/E analysis seeks to determine whether the occurrence of an event after vaccination (“the observed”) is more frequent than the “expected” occurrence of the same event in the absence of exposure to the vaccine of interest. The methodology and recommendations for O/E analyses in safety surveillance have been comprehensively summarized by Mahaux et al. (Mahaux et al., 2016).

The initial steps for O/E analysis involve obtaining the best possible estimates for the numerator and the denominator in the O/E ratio. The numerator (i.e., the “observed” component in a real-world setting) corresponds to the count of a specific AE reported spontaneously, or voluntarily, by individuals after vaccination. AEs, including AESIs, may be reported to MAHs and/or regulatory authorities by any individual, whether or not they are a healthcare professional. The reported AEs are coded using the Medical Dictionary for Regulatory Activities (MedDRA) and stored in a safety database, such as the MAH’s internal safety database or a regulatory authority safety database, such as EudraVigilance in the EU or the Vaccine Adverse Event Reporting System (VAERS) in the US, which is then used by the MAHs and/or regulatory authorities to conduct pharmacovigilance activities. MedDRA-based search strategies are used to query the safety databases and retrieve the ICSRs for each AESI. Using ICSRs from spontaneous reporting databases as the numerator in the O/E calculation relies on robust reporting, collection, and documentation of AEs from vaccinated individuals.

The denominator (i.e., the “expected” rate) is calculated using three different components: exposure data (e.g., number of specific vaccine doses administered to the population, either estimated or actual), the postvaccination timeframe an event is likely to occur (risk window [RW]), and the background incidence rate (BIR) of the AESI in the unexposed target population. Determining the values of the numerator and denominator is crucial and must closely match the true values, as their accuracy (or lack thereof) directly impacts the O/E analysis results and their interpretation.

## 3 AESIs and frequency of O/E analyses

Identifying AESIs for high priority monitoring was a key challenge for a planned mass vaccination campaign. The Brighton Collaboration (BC), an independent group that facilitates standardization of vaccine safety surveillance, supported the Safety Platform for Emergency vACcines (SPEAC) project to develop a COVID-19 vaccine AESI list. This work was part of BC’s support for the safety assessment of vaccine development funded by the Coalition for Epidemic Preparedness (CEPI). The BC-SPEAC AESI list, publicly available to MAHs and regulatory authorities, included events relevant to vaccination in general, specific to COVID-19 vaccine platforms, or relevant to COVID-19 infection itself. Of note, some AESIs were “theoretical concerns” (Safety Platform for Emergency vACcines, 2020). Other AESI lists were developed by the US CDC, the VACcine COVID-19 monitoring readinESS (ACCESS) project (funded by EMA), the Global Vaccine Data Network (GVDN) and by regulatory authorities including the US FDA, United Kingdom MHRA, Australia TGA, and New Zealand Medsafe. While these AESIs lists were similar, they were not identical, and their operationalization among MAHs varied (Black et al., 2021).

As per the “EMA Consideration on core requirements for RMPs of COVID-19 vaccines” (European Medicines Agency, 2022a), MAHs were required to submit frequent Summary Safety Reports (SSRs)^3^ to EMA for prompt review of COVID-19 vaccine safety profiles and detection of safety information, including new signals. O/E analyses were required to be included in the SSRs, along with evaluations of new safety information, information on safety concerns documented in COVID-19 vaccine EU-risk-management plans (EU-RMPs), reviews of signals identified during the reporting period, and any additional safety topics requested by EMA and/or other regulatory authorities.

For the first year following initial COVID-19 vaccine authorization for all COVID-19 vaccine MAHs, and longer for some, SSRs were produced monthly and submitted to EMA within 15 days according to an agreed schedule (typically, the data-lock point was the last day of the month and the submission of the SSR occurred on the 15th of the following month). These reports preceded and complemented the submission of Periodic Safety Update Reports (PSURs) as per EU GVP Guidance on Vaccines (European Medicines Agency, 2013). Many regulatory authorities around the world adopted SSRs, each requesting tailored reports meeting their specific data requirements, schedules, and assessment timetable. Beyond scheduled O/E analyses required by EMA for SSRs and PSURs (European Medicines Agency, 2021a), numerous *ad hoc* O/E analyses were performed in response to specific requests from EMA and other regulatory authorities. The inherent complexity coupled with the required frequency of conducting these O/E analyses presented significant challenges for COVID-19 vaccine MAHs.

## 4 COVID-19 specific challenges and limitations

### 4.1 Challenges in estimating the numerator/“observed” counts

The anticipated substantial volume of ICSRs was a sound rationale for using O/E analyses to support robust signal detection with a rapid turnaround time. However, challenges emerged in determining the most accurate assumptions for the numerator to ensure it best corresponded to the number of reported cases for a defined AESI.

#### 4.1.1 Inclusion of unadjudicated cases

Adverse Events following vaccination were primarily reported to regulatory authorities, who subsequently forwarded them to the respective MAHs for uploading into their safety databases. As regulatory authorities were the primary recipient of most ICSRs, MAHs were unable to collect follow-up data for a large proportion of cases due to practical reasons. This lack of follow-up, combined with the large volume of ICSRs and tight case processing timelines, prevented MAHs from adjudicating the AESI reports to determine their medical consistency with true AESI cases before inclusion in O/E analyses. Therefore, MAHs adopted a conservative approach and, without adjudication, included in the numerator all cases that were retrieved by the MedDRA-based search strategies. This approach diverged from the preferable practice of adjudicating AE cases using established AESI case definitions before including them as cases in the O/E analyses numerator. The inclusion of unadjudicated^4^ cases likely led to an overestimation of AE counts and introduction of potential bias by incorporating cases that may not have met validated AESI criteria.

#### 4.1.2 Potential biases in spontaneous reporting: over/under reporting and duplicate ICSRs

Ideally, post-marketing surveillance activities, which rely on well documented ICSRs, confirm the safety profile established during clinical development and allow for the detection of any new or rare potential safety signals arising when a larger population is exposed. Underreporting is a well-documented limitation of spontaneous reporting systems (Clothier et al., 2014). Considering the unprecedented call for COVID-19 post-vaccination AE reporting and vigilance for safety issues, it may not be accurate to assume that all new potential reactions of clinical importance were widely underreported. COVID-19 pandemic and vaccine news and updates were continuously disseminated through physical, digital, and social media outlets, reaching millions around the world on a daily basis. Indeed, information about COVID-19 was endlessly updated and available for the consumption of global public health officials, medical professionals, and the general public. The saturation of both lay and medical communication channels with COVID-19 information, and then with COVID-19 vaccine information, served as a constant reminder to report AEs in vaccinees, contributing to stimulated reporting. The high degree of transparency with which signals and potential signals were addressed between MAHs and regulatory authorities, along with regular public communications about safety by regulators to maintain public trust, may have further increased media coverage and stimulated AE reporting.

Additional factors possibly biasing AE reporting included differences in national vaccination prioritization strategies across countries (e.g., elderly in long-term care facilities, healthcare workers, immunocompromised individuals), regional variations in pharmacovigilance infrastructure capabilities, and different reporting approaches, with some countries proactively soliciting AE data from vaccinees, while others collected AEs passively, potentially skewing reporting from certain populations or regions.

Potential inconsistencies in AE coding conventions used across regulatory authority safety databases and the absence of standardized MedDRA search strategies for AESIs among MAHs may have led to over- or undercounting of cases in the numerator, depending on whether broad/sensitive or narrow/specific search strategies were selected. This lack of harmonization also contributed to difficulties in de-duplicating ICSRs at the time of O/E analysis for the “numerator” component.

#### 4.1.3 Missing data and subsequent assumptions

In addition to the limitations described above, most ICSRs lacked data on one or more key elements such as age, sex, dose, or time to onset (TTO) of events. MAHs chose to retain these cases in the O/E analyses by including all cases with missing TTO in the observed counts across all RWs, and/or by applying imputations based on the distribution of cases with known TTO, as per Mahaux et al.’s methodology (Mahaux et al., 2016). This conservative approach aimed at minimizing the risk of missing a signal that would need further investigation. In a similarly conservative manner, reported cases were categorized by age, sex, and dose, with unknown values either excluded from the analysis or proportionally distributed across groups based on cases with complete information. These imputation methods, utilized by some MAHs, might have led to some degree of over- or under-estimation of the O/E analyses for specific strata requested by the regulatory authorities for several AESIs/safety topics.

### 4.2 Challenges in estimating the denominator/“expected” counts

The accurate determination of the three components used in the denominator of O/E analyses (BIR, exposure data, and the RW) also presented challenges, as described below.

#### 4.2.1 Background incidence rate selection

A critical component in calculating expected AESI numbers is the availability of reliable BIRs for specific events. Traditionally, BIR estimates are retrieved from published population-based epidemiological research (Mahaux et al., 2016; Black et al., 2021; Esposito et al., 2018; Klein et al., 2010; Willame et al., 2021a). During the COVID-19 pandemic, several initiatives (e.g., ACCESS, GVDN, Observational Health Data Sciences and Informatics (OHDSI), The Biologics Effectiveness and Safety Initiative [BEST]) generated BIRs from electronic healthcare databases from different countries, with results becoming available progressively throughout the pandemic (Willame et al., 2021b; Phillips et al., 2023; Moll et al., 2023). However, the delayed availability of these data complicated their utilization by MAHs. Further, not all published estimates were immediately suitable for use in O/E analyses. Occasionally, BIR data from some initiatives were presented in graphs without accessible numeric values, or the 95% confidence intervals were not provided (Kurata et al., 2011). More recent initiatives have included estimate tables in annexes, facilitating easier and wider use (Willame et al., 2021b; Li et al., 2021; Nasreen et al., 2022).

For some AESIs, the abundance of available estimated BIRs presented methodological challenges. Significant heterogeneity was observed across data source types (e.g., claims, electronic health records), within the same data source, and/or across time periods, complicating the selection of the most appropriate BIR. The lack of standardized AESI definitions hindered consistent evaluation across studies (Gandhi-Banga et al., 2025). For certain AESIs, insufficient data stratification limited the usefulness of applying these rates to age- and gender-specific O/E analyses. In addition, age groups were not harmonized, with some estimates provided in 5-year bands and others in 10-year bands, not in alignment with COVID-19 vaccination-approved populations (e.g., 6–12 years, 12–16 years, etc.).

Newly emerging AESIs, such as Thrombosis with Thrombocytopenia Syndrome (TTS) and conditions such as Multisystem Inflammatory Syndrome (MIS), lacked established BIRs. Composite outcomes or symptom-based conditions without specific diagnostic codes in clinical practice (e.g., encephalomyelitis, eye disorders, dysgeusia, menstrual disorders) posed additional challenges for identifying appropriate data in healthcare databases and estimating BIRs. The following two examples illustrate AESIs that were not suitable for O/E analyses while they were required:

- MIS was a recently described severe inflammatory condition affecting multiple organ systems following infection or immune stimulation by SARS-CoV-2. To screen potential MIS cases in spontaneous reporting systems, MedDRA Preferred Terms (PTs) for conditions potentially associated with MIS were applied. At the request of a regulatory authority to a MAH, a more conservative search strategy was used to include adjacent terms such as sepsis. However, MIS and sepsis BIRs differ substantially in the general population. MIS is a rare condition, with an estimated incidence of 0.5–2.0 per 100,000 person-years in children following SARS-CoV-2 infection. In contrast, sepsis is far more common, with an estimated incidence of 300–600 per 100,000 person-years in the general adult population, and even higher rates in neonates and older adults. This substantial baseline rate difference rendered O/E analyses of MIS uninterpretable, since results were confounded by the inclusion of conditions with distinct epidemiological profiles and clinical presentations. Of note, the eventual inclusion of MIS in MedDRA and medical coding systems, along with increased physician familiarity, should improve O/E analyses on MIS in the future.
- Composite condition AESIs, such as encephalomyelitis, also presented significant challenges due to their constituent disorder heterogeneity, each with distinct BIRs. Encephalomyelitis, for example, includes a range of inflammatory conditions affecting the brain and spinal cord, such as acute disseminated encephalomyelitis (ADEM) and infectious or autoimmune encephalomyelitis, each with different baseline frequencies in the general population. Even when an overall incidence estimate for a similar composite term exists, there is no guarantee that the relative prevalence of each component remains consistent between observed cases and the expected population in the reference source. If a relatively rare component within the composite is imbalanced in the observed cases but the more common components are less frequent in the observed cases, a signal could easily be masked within the aggregate estimate. Consequently, using composite terms in O/E analyses may obscure meaningful results.

Additionally, some regulatory authorities requested O/E analyses to assess possible recurrence, exacerbation, or worsening of pre-existing autoimmune or neuroinflammatory conditions. Providing analyses for these requests was challenging, as BIRs in populations with underlying conditions were largely unavailable.

Finally, consensus or guidance among stakeholders on BIR selection best practices would have been beneficial. Indeed, the absence of comprehensive BIR selection guidelines led to wide variation in methodological choices and inconsistent approaches among stakeholders (MAHs, health authorities, and regulatory authorities), making it difficult to meaningfully compare and interpret O/E results across time, geography, populations, and vaccine formulations.

#### 4.2.2 Estimation of global exposure

Estimation of an accurate denominator for O/E analyses also depends on the availability of reliable exposure data (i.e., the number of specific vaccine doses administered over time by country, MAH, age, and sex of vaccinees). Ideally, the actual number of vaccine doses administered would be used to calculate the expected cases in the denominator. However, an estimate of the administered doses is often impossible to obtain, requiring proxies such as doses distributed or sold. These proxies can be inaccurate since only a portion of distributed doses may be administered to patients. During the pandemic, some government agencies made COVID-19 vaccine administration data publicly available, increasing confidence in exposure estimates. However, these data were available to MAHs according to a timeline and in a structure determined by the respective agencies. The US Centers for Disease Control and Prevention (CDC) and the European Centre for Disease Prevention and Control (ECDC) provided data on COVID-19 vaccine doses administered in the US and EU/European Economic Area (EEA) countries, respectively, stratified by MAH. The publicly available US CDC exposure data also included data on the age and sex of vaccinees. In contrast, EU data accessibility varied by country, with only overall counts or age-stratified data accessible to MAHs. When administration with an unspecified brand of COVID-19 vaccine was reported, some MAHs estimated distribution by applying the same proportional distribution of known vaccines. Although age-stratified exposure data were available for some countries, the age categorization did not fully match the BIR stratification, necessitating methodological adjustments to ensure valid comparative analyses. Where detailed age-stratified data were unavailable for specific countries, information from other EU countries with more complete data was used to impute the distribution of administered doses. Alternatively, some MAHs applied Mahaux et al.’s approach, using observed data’s age/sex stratification to estimate the age/sex stratification of the administered doses (Mahaux et al., 2016).

COVID-19 vaccine administration data were systematically updated by both the CDC and ECDC in the 2020–2023 period. These data underwent retrospective corrections, which complicated trend interpretation. The US ceased public data updates on 11 May 2023, after the WHO ended the state of emergency on 5 May 2023 (World Health Organization, 2025). Shortly afterwards, the ECDC also stopped their public updates of exposure data. Regulatory agencies continued periodic and *ad hoc* O/E requests to MAHs, who were faced with the choice of using inaccurate exposure data or conducting analyses with cutoff dates driven by the most recent CDC and/or ECDC exposure data updates (thus excluding newer spontaneous reports). Some MAHs preferred cutting off analyses at the most recent exposure data update to maintain O/E calculation accuracy and consistency. Others reverted to traditional methods using claims data and/or distribution data, making interpretation of the results challenging due to the introduction of significant uncertainty and variability. Besides requiring strong assumptions about the proportion of distributed doses administered, estimations based on distribution lacked the critical vaccinee demographic characteristics needed for appropriate background rate assignment, which weakened the reliability of analyses. Use of administrative healthcare data offered a potential solution in the US (accepting limited national-level vaccine administration capture outside of pharmacy settings), but this did not facilitate analysis of data at the global level. Extending public availability of high-quality exposure data would have better supported ongoing pharmacovigilance activities.

Outside the US and EU, exposure data availability, timeliness and content varied, limiting the feasibility of regionally tailored analyses, with some MAHs using US and EU data to extrapolate the number of administered doses in other regions. While data catalogs like Our World in Data offered useful overviews of global trends, they often lacked critical granularity, such as brand-specific information, limiting their utility for more detailed analyses (Our World in Data).

#### 4.2.3 Selection of risk windows

The RW, which defines the presumed period of increased vaccine-associated risk for a specific AESI after vaccine administration, is used in both numerator and denominator calculations. Selecting an appropriate RW is crucial for avoiding misclassification bias. RWs are typically defined based on previous publications or clinical expertise. An alternative approach involves analyzing the TTO distribution of the relevant ICSRs to determine when most events occurred (Gordillo-Marañón et al., 2024). However, uncertainties on RW selection may exist for some events, particularly for new conditions. In March 2021, the AESI Working Group of Vaccines Europe, which represents European vaccine companies and includes safety clinicians and epidemiologists from different MAHs, met to define primary and secondary RW for certain AESIs based on their extensive experience with previous vaccines. Despite this non-competitive collaborative initiative and the examination of identical AESIs, significant variability was observed in RW definitions used in O/E analyses performed by various stakeholders (MAHs, health authorities, and regulatory authorities). This variability potentially resulted in under- or over-estimation of O/E results. To address RW uncertainty, multiple RWs, including shorter (7- or 14-days) and longer (21- or 42-days) TTOs were included in the MAH’s sensitivity analyses. This highlights, as for BIR selection, the need for RW harmonization, as results and conclusions may be impacted.

### 4.3 Overall challenges

#### 4.3.1 O/E analyses in specific population

O/E analysis was not feasible for certain AESIs. For pregnancy-related AESIs, O/E could not be performed due to the inability to determine relevant exposure and RWs. Similarly, O/E analysis was not possible for AESIs without an unexposed comparator, such as vaccine-associated enhanced disease.

#### 4.3.2 Differences in coding dictionaries

While not specific to the COVID-19 context, the use of different dictionaries for AESI coding introduced potential inconsistencies between cases in the numerator and BIRs in the denominator. MedDRA is used for coding AEs in reporting safety databases while International Classification of Diseases (ICD) coding is generally used in medical databases used to estimate BIRs. At times, the coding of conditions in dictionaries is not sufficiently similar.

#### 4.3.3 Interval *versus* cumulative analyses

According to the second version of the EMA core RMP requirement (V2.0) (European Medicines Agency, 2022a), O/E analyses were expected for both interval and cumulative ICSRs to monitor temporal trends and facilitate the identification of emergent safety signals. However, this introduced several interpretational challenges. Specifically, the grouping of cases received within discrete monthly periods did not ensure all events were linked to vaccinations occurring within that interval, particularly when initial reporting was delayed or regulatory authorities experienced lags in AE report processing. Additionally, the characteristics of vaccinee populations evolved rapidly during the early vaccination campaign phases, complicating the reliability of cross-interval comparisons. Moreover, even during extensive vaccination efforts, rare AESIs over short time periods produced reporting rates with wide confidence intervals, severely limiting interpretability of informal comparisons between interval and cumulative analyses. Some MAHs successfully challenged the value of interval analyses, arguing that cumulative analysis provided a more comprehensive picture of safety trends with better statistical reliability. However, at least one MAH was still required to perform interval O/E analyses for almost a full year post-authorization as part of monthly SSRs.

#### 4.3.4 Sensitivity analyses to assess uncertainties

Despite the apparent numerical precision of O/E ratios, it is important to acknowledge that these values are derived from multiple underlying assumptions and estimations, resulting in uncertainties. To mitigate those, MAHs performed sensitivity analyses to test assumptions and evaluate their impact on results and conclusions. Table 1 provides examples of sensitivity analyses performed by MAHs. To avoid missing any cases, MAHs conducted O/E analyses using both broad (sensitive) and narrow (specific) AESI search strategies. To account for potential underreporting, sensitivity analyses were performed using at minimum two different reporting rates: 50% and 25% (a 50% reporting rate assumes half of AESI occurrences were reported, while a 25% rate assumes only one-quarter of the cases were reported). To evaluate denominator assumptions, sensitivity analyses were conducted using varying BIR estimates (low and high values) for specific AESIs when possible. Similarly, when RW uncertainties existed, additional sensitivity analyses were conducted using secondary or tertiary RWs.

**TABLE 1:** Type of sensitivity analyses performed per region for each AESI.

Per AESI and region	Parameters	Type of sensitivity analyses (in addition to base analyses)
Numerator: “Observed” count	Case definition	• Broad/Narrow definition
	Adjudication	• Cases meeting a case definition (adjudicated cases)
	Processed cases	• Processed cases + backlog cases
	Reporting rate	• 50% Reporting rate • 25% Reporting rate • Other reporting rate
	Time to onset	• Cases with missing time to onset
Denominator: “Expected” count	BIR	• BIR mid-range • BIR per age group • BIR per age group and sex
	RW	• Secondary RW • Additional RW(s)
	Doses administered	• Dose number • Formulation (after authorization of variant-adapted vaccines)

Abbreviations: AESI, adverse events of special interest; BIR, background incidence rates; RW, risk window.

In addition, as the pandemic evolved, and vaccination schedules became more complex, additional analyses were required to stratify analyses by dose number and vaccine formulation. Consequently, MAHs faced hundreds of O/E ratios to produce and analyze, often yielding inconsistent results.

Sensitivity analyses allowed MAHs to implement approaches that were sufficiently conservative without being excessively restrictive. In fact, the cumulative effect of multiple conservative assumptions could result in risk overestimation, while insufficient conservatism could lead to risk underestimation, both scenarios potentially compromising the validity of O/E analyses. Finding a balance between minimizing false safety signals while maintaining sensitivity for detecting true safety signals was crucial. While O/E analysis focused on specific AESIs, it did not eliminate the possibility that unexpected AEs which would have not been identified by this methodology might occur. In addition, some AESIs could not be effectively identified through O/E signal detection due to insufficient reliable data.

#### 4.3.5 Volume of O/E analyses

The high volume of O/E calculations presented a challenge, and the list of AESIs requiring O/E analyses by MAHs evolved over time. In early 2021, at the start of post-authorization pharmacovigilance, monthly SSRs of some MAHs included O/E analyses on upwards of 45 different AESIs (leading to approximately 300 O/E estimates with the sensitivity analyses) detailed over thousands of pages in the report annexes. Over time, AESIs were progressively added and by the end of 2023, some MAHs were conducting O/E analyses on over 60 AESIs. Sensitivity analyses expanded as well; additional dose numbers and formulations led to additional stratifications. As knowledge advanced, deeper analyses into certain AESIs were conducted, including customized age categories and RW, stratification by sex, nuanced case definitions (narrow and broad, use of BC case definition criteria), and O/E analyses with a range of BIRs. By late 2023, the volume of O/E point estimates had doubled (>600 estimates) compared to early reports. Due to lack of regulatory guidance on post-authorization timeframes for O/E analyses and transition points to other surveillance methods, MAHs adopted a conservative approach that resulted in an expanding volume of analyses over time.

In addition to the rapid-turnaround monthly SSRs and bi-annual PSURs required by EMA and other regulatory authorities, MAHs continuously received *ad hoc* requests for additional safety evaluations. On average, two to five additional O/E analyses as part of signal assessment investigations were conducted per month during the 2021–2023 period. These were typically requested with a short turnaround time (e.g., one month) and entailed in-depth investigations of potential cases, separate literature reviews to identify supplementary vaccine safety information and BIRs, statistical analyses to produce O/E ratios and confidence intervals, and production of a cohesive report considering the totality of all available data. This volume of work required dedicated teams of clinicians, scientists, epidemiologists, and statistical programmers.

## 5 Relevance of O/E analyses during the COVID-19 pandemic–lessons learned

### 5.1 Resource utilization

O/E analyses required significant dedication of cross-functional resources and expertise to meet the expectations of regulatory authorities. Initial work included decisions on the AESIs to be monitored (considering the multiple lists from vaccine groups and various regulatory authorities) and translation/operationalization of AESI concepts to MedDRA PTs for the appropriate identification of ICSRs in the safety database. Additionally, for BIRs that were not available from the ACCESS project, translation of AESIs to International Classification of Diseases, 10th Revision (ICD-10) codes was necessary for appropriate data identification. The MedDRA based search strategies for each AESI required continuous monitoring by the MAHs and updating due to the standard twice-yearly MedDRA updates, regulatory authority requests, or when considered appropriate due to evolving vaccine safety profiles. Further expertise was needed to address missing, incomplete, or poor-quality data and to collect all the data (e.g., exposure data, observed cases) needed for the hundreds of O/E analyses. Cross-functional expertise was required to conduct O/E analyses and also to interpret the data contextually, weighing evidence regarding potential signals and their causal association with vaccination.

While this challenge has been particularly relevant for MAHs, regulatory authorities may likewise have encountered similar complexities in reviewing and interpreting extensive data from several MAHs within constrained timelines. The allocation of extensive resources to complex O/E analyses and including them in SSRs may have inadvertently diverted attention from other effective safety surveillance approaches, such as case reviews, trend analyses, and literature evaluation, which have consistently demonstrated their value in identifying potential safety concerns.

Considering the time and resources available for pharmacovigilance activities across stakeholders, it is worthwhile to reconsider whether the focus on using O/E analyses for surveillance of a large group of mainly theoretical concerns, instead of on other incoming safety information, was the best use of resources in monitoring new vaccine safety profiles and protecting public health.

### 5.2 O/E analyses for signal detection

Review of the EMA-Pharmacovigilance Risk Assessment Committee (PRAC) meeting minutes during 2021 (when COVID-19 vaccines first became available) provides an informative look at the earliest EMA PRAC-identified safety signals^5^ and their sources (European Medicines Agency, 2022b; European Medicines Agency, 2022c; European Medicines Agency, 2021b; European Medicines Agency, 2021c; European Medicines Agency, 2021d; European Medicines Agency, 2021f; European Medicines Agency, 2021g; European Medicines Agency, 2021h). PRAC is EMA’s committee for assessing safety and making pharmacovigilance recommendations based on its assessments. Some 2021 PRAC-identified signals were prespecified as AESIs in the MAHs’ AESI lists (e.g., erythema multiforme) while others were not (e.g., localized swelling with dermal filler injections, TTS). Regardless, none of the events during this time period were identified as signals based on O/E analyses as a signal detection method (Caplanusi et al., 2024). Indeed, all signals arose from traditional safety surveillance activities, i.e., from individual experiences communicated (mainly via ICSRs) to MAHs or regulatory authorities and noted as deserving further evaluation.

The results of MAHs O/E analyses for signal detection did yield some elevated numerical ratios (ratio >1). However, when this “numerical signaling” prompted further assessment of an AESI, none were subsequently confirmed to be causally associated with vaccination. Given the extensive number of calculations generated across and within AESIs, some spurious O/E elevations without clinical significance were inevitably observed. The high sensitivity and low specificity of O/E in this context can lead to false positives, impacting decision-making and resource allocation.

### 5.3 O/E analyses for signal refinement

O/E analyses have traditionally been used to refine the understanding of a safety signal after its identification from other sources or methodologies. A similar experience occurred with COVID-19 vaccines, with myocarditis being an example. Myocarditis was included in the initial AESIs list (Safety Platform for Emergency vACcines, 2020) and was subject to O/E analyses from the outset. However, the initial safety signal was not detected through this quantitative method. Instead, it was identified via conventional pharmacovigilance practices. Specifically, the Israeli Ministry of Health noted cases of myocarditis in young males shortly after they received the Pfizer-BioNTech mRNA COVID-19 vaccine (Reuters, 2021). Myocarditis was subsequently flagged as a signal by EMA, who requested further evaluation from the MAHs of mRNA COVID-19 vaccines, including additional stratified O/E analyses. While initial O/E signal detection analyses had not shown an elevated ratio, age and sex-specific analyses revealed statistically significant O/E ratios in younger individuals, particularly males, and following the second dose, as reported by the Israeli Ministry of Health and EMA (European Medicines Agency, 2021; European Medicines Agency, 2021e). These findings, in the context of the totality of available safety data, prompted an update to the product information to alert healthcare professionals and patients.

In addition to its important role in signal strengthening for myocarditis/pericarditis, O/E analyses were also key in refining the signal of TTS by EMA. Access to additional stratified exposure data made available to EMA by member states enhanced the health authority’s ability to investigate potential at-risk subpopulations (Gordillo-Marañón et al., 2024; Durand et al., 2023).

Van der Boom’s publication provides another example of how O/E analyses were employed to refine a safety signal (van der Boom et al., 2023). During the pivotal phase 3 clinical trials of mRNA COVID-19 vaccines, several cases of Bell’s palsy, a facial paralysis, were reported among vaccine recipients (Baden et al., 2021). Consequently, this outcome was closely monitored as an AESI during post-marketing surveillance for all COVID-19 vaccines. The Netherlands Pharmacovigilance Centre (Lareb) conducted O/E analyses to explore the Bell’s palsy signal in greater depth to identify potentially at-risk subpopulations. The observed cases consisted solely of medically confirmed Bell’s palsy, which represents a key distinction from the broader approach used in O/E analyses for signal detection. This analysis led to an update of the Summary of Product Characteristics (SmPC) for one of the two adenovirus-vectored COVID-19 vaccines, which, unlike the other marketed COVID-19 vaccines at that time, had not listed facial paralysis as an adverse drug reaction (ADR) (van der Boom et al., 2023).

Overall, O/E analyses, as traditionally applied in signal refinement, were proven to be of value in these situations.

## 6 Discussion

The COVID-19 pandemic, declared in March 2020, emerged with no established treatments or vaccines available and limited disease knowledge. COVID-19 vaccines were developed and authorized at unprecedented speed, making rapid detection, assessment, and communication of safety information essential for guiding policy decisions and maintaining public confidence.

Routine signal detection commonly relies on quantitative methods such as disproportionality analysis (DPA), which assesses the frequency of reported AEs for a given product relative to the overall reported AEs in a reference safety database. Commonly used statistical measures include the Proportional Reporting Ratio (PRR), Reporting Odds Ratio (ROR), and Empirical Bayes Geometric Mean (EBGM). In addition to those methods and as per the “EMA Consideration on core requirements for RMPs of COVID-19 vaccines”, EMA requested that MAHs conducted O/E analyses as an unconventional quantitative method for signal detection (European Medicines Agency, 2022a; Durand et al., 2023). Typically, O/E analysis is a tool for signal refinement, i.e., a method to evaluate a safety signal identified through other pharmacovigilance methods (Mahaux et al., 2016), with the important caveat that O/E analyses must be “based on good-quality data” (European Medicines Agency, 2013). As highlighted in a recent publication, the pandemic-specific use of O/E for signal detection transformed its primary nature from one based on medical expertise and epidemiology to a more algorithmic approach resembling DPA, a type of O/E that differs in how the way the expected component is calculated (Van Holle, 2025).

The accuracy of O/E analyses heavily depends on the quality and reliability of parameters used in the numerator and denominator. In addition, the value of O/E analyses in safety decisions must be weighed in the context of the totality of safety data analyzed, neither overstating nor underestimating their importance.

The potential value of these analyses could be highest at the start of the mass vaccination campaigns when unprecedented high volumes of ICSRs are received in a relatively short time, a scenario very atypical compared to the volume of ICSRs usually received following the approval of a new vaccine. As the safety profiles of COVID-19 vaccines became well-characterized and the volume of ICSRs decreased over time, the usefulness of O/E analyses for signal detection decreased, creating an opportunity to feasibly implement more effective pharmacovigilance methods for assessing incoming safety data.

There is an active discussion among regulatory authorities about the future direction of O/E analysis and its role in routine pharmacovigilance activities. In a post-pandemic review, EMA stated that “while O/E analysis is viewed as a supportive tool that can facilitate contextualization of data during extreme circumstances, its limitations must be considered on a case-by-case basis” (European Medicines Agency, 2023).

From an industry perspective, the development of methodological best practices through a common protocol, similar to that discussed by an International Coalition of Medicines Regulatory Authorities working group for real-world evidence (Beck et al., 2025), would strengthen and harmonize practices. Such an approach would not only facilitate analysis and allow comparability between results generated from different MAHs, but would ultimately optimize the benefit/risk assessment of available vaccines and contribute to public health. A collaborative approach, developed through a multistakeholder expert group including regulatory authorities and industry partners, represents the critical path forward to align on the optimal use of O/E analysis and establish a shared understanding of results interpretation and methodological limitations. This collaborative network must address when O/E analysis is most appropriate and when its utility is limited, such as for more common events or those already identified as risks or further characterized by robust real-world evidence. Furthermore, a consensus-building through a public-private partnership on parameters such as (i) the minimum number of observed cases required (e.g., overall and strata-specific with age/sex stratified analyses); (ii) the case definitions and corresponding search strategies (MedDRA and ICD codes, as applicable); (iii) the generation, selection and use of BIRs; (iv) the definition of the RW; and (v) the sources of exposure data (stratified by age, gender, dose, and vaccine brand) is essential.

Lessons learned by COVID-19 vaccine MAHs during the pandemic and other stakeholders support the fact that O/E analyses deliver maximum value when focused on signal refinement rather than signal detection. We must acknowledge that O/E analyses relevance are context-dependent and offers meaningful results when critically assessed for the specific topic under evaluation. This perspective would represent a more efficient allocation of resources and help to ensure that O/E analyses effectively contribute to the continuous benefit-risk assessment of authorized vaccines.

## Footnotes

1 ICSR (synonym: AE report) refers to an adverse event(s) in an individual following treatment or vaccination. Adverse events reported are entered into the MAHs safety reporting database as unique reports. They are not necessarily causally associated with the product.

2 AESIs refer to adverse events or medical conditions identified for focused safety surveillance by regulatory authorities, MAHs or safety advisory bodies. When an AESI is reported in an ICSR (AE report), the information provided is not always sufficient to confirm that the event is truly representative of the AESI diagnosis.

3 Referred to as monthly summary safety reports (MSSR) by EMA.

4 Adjudicated reports refer to ICSRs that were individually medically reviewed by the MAHs and assessed to be consistent with the AESI diagnosis.

5 Localized swelling with dermal filler injections, Immune thrombocytopenia, Embolic and thrombotic events, Capillary leak syndrome, Acute macular outer retinopathy, Myocarditis and pericarditis, Erythema multiforme, Glomerulonephritis and nephrotic syndrome, Multisystemic inflammatory syndrome in children, Autoimmune hepatitis (Source: minutes of EMA PRAC meetings from 08 March 2021 through 02 December 2021).
